# Annexin A1 in Alzheimer’s disease: A new therapeutic strategy focusing on neuroinflammation

**DOI:** 10.4103/NRR.NRR-D-25-00505

**Published:** 2025-09-03

**Authors:** Luiz Philipe de Souza Ferreira, Cláudia A. Valente, Cristiane D. Gil

**Affiliations:** Structural and Functional Biology Graduate Program, Escola Paulista de Medicina, Universidade Federal de São Paulo (UNIFESP), São Paulo, SP, Brazil; Instituto de Farmacologia e Neurociências, Faculdade de Medicina, Universidade de Lisboa, Lisboa, Portugal

Neurodegenerative diseases affect millions of people worldwide, with Alzheimer’s disease (AD) being the leading cause of dementia. It is estimated that more than 50 million people live with this condition, which is expected to triple by 2050, driven mainly by the aging of the global population (GBD 2019 Dementia Forecasting Collaborators, 2022). The pathogenesis of AD is characterized by a complex interplay of genetic, molecular, and cellular mechanisms, particularly involving amyloid-beta (Aβ) and Tau proteins. These processes trigger the reactivity of glial cells, which in turn contributes to the degeneration of hippocampal neurons and a significant reduction in synapse density (Van Zeller et al., 2021).

**Figure 1:**
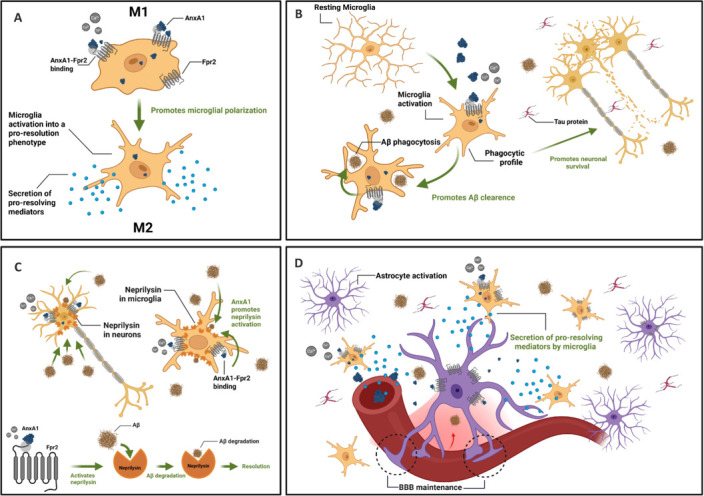
Annexin A1 is a regulator of neuroinflammation in Alzheimer’s disease. (A) During neuroinflammation, AnxA1 modulates microglial polarization by promoting the transition from a pro-inflammatory M1 phenotype to a pro-resolving M2 state via interaction with the FPR2. (B) In response to Aβ and Tau protein deposition, AnxA1 enhances Aβ uptake by microglia, contributing to its clearance from the microenvironment. (C) AnxA1 also promotes Aβ clearance by upregulating neprilysin expression in neurons, thereby facilitating Aβ peptide degradation. (D) By modulating microglia phenotype, AnxA1 also mitigates astrocyte activation, thereby preserving the integrity of the BBB, which is compromised by Aβ and Tau accumulation. Created with BioRender.com. Aβ: Amyloid-beta; AnxA1: annexin A1; BBB: blood–brain barrier; FPR2: formyl peptide receptor 2; M1: pro-inflammatory microglia phenotype; M2: anti-inflammatory/pro-resolving microglia phenotype; Tau: hyperphosphorylated Tau protein.

The search for new therapeutic strategies for AD has instigated researchers worldwide to explore new strategies offering greater efficacy and fewer side effects, particularly targeting neurons and glia. Annexin A1 (AnxA1), a 37 kDa Ca^2+^-dependent phospholipid-binding protein, has emerged as a promising candidate for mitigating neuronal death and glial toxicity by initiating a cascade of events that promotes a pro-resolving response (**Figure 1**), primarily mediated by microglia and astrocytes, as detailed previously (de Souza Ferreira et al., 2025). Studies have reported altered AnxA1 expression in AD: while AnxA1 is upregulated in the brain and microglia surrounding Aβ plaques (McArthur et al., 2010), its levels are reduced in the serum of AD patients (Park et al., 2017), suggesting compartment-specific regulation. Moreover, AnxA1 can be cleaved by proteases (elastases, metalloproteases, among others) into an inactive 33 kDa fragment, which limits its beneficial actions. Indeed, elevated levels of cleaved AnxA1 were observed in neurodegenerative diseases, particularly AD and dementia with Lewy bodies, and were associated with amyloid load, inflammation, and apoptotic markers (Chua et al., 2022).

Through its interaction with the formyl peptide receptor 2 (FPR2), AnxA1 activates the AMP-activated protein kinase-mammalian target of rapamycin (AMPK-mTOR) pathway, shifting microglia from a pro-inflammatory phenotype to a pro-resolving one, thereby reducing neuroinflammation and neuronal damage (Xu et al., 2021). Additionally, AnxA1 modulates the CX3CR1-CX3CL1 axis, facilitating microglia-neuron interactions essential for synapse formation and cognitive restoration (Zheng et al., 2022). In astrocytes, AnxA1 plays a critical role in tissue repair during blood-brain barrier breakdown. Furthermore, AnxA1 contributes to the maintenance of blood-brain barrier integrity by binding to FPR2 expressed on brain endothelial cells, where it inhibits RhoA signaling, stabilizes the cytoskeleton, and decreases paracellular permeability (Cristante et al., 2013). Treatment with AnxA1 directly targets reactive astrocytes via the MAPK pathway, inhibiting p38 and JNK activation, and fostering a pro-resolving response by suppressing the release of pro-inflammatory mediators such as interleukin-1β, tumor necrosis factor-α, and monocyte chemoattractant protein-1 (Luo et al., 2020).

Another key function of AnxA1 is its role in promoting the selective clearance of apoptotic neurons by microglia, thereby maintaining tissue homeostasis. Microglia-derived AnxA1 facilitates the recognition and phagocytosis of apoptotic neurons by forming a bridge between dying neurons and microglia through FPR2 signaling (McArthur et al., 2010). Additionally, AnxA1 contributes to Aβ clearance through two distinct mechanisms: it promotes neprilysin expression in neurons, a membrane zinc metalloprotease that is capable of digesting Aβ peptide, reducing the amyloid load within the brain, and increases Aβ uptake by microglia, facilitating its phagocytosis. Beyond Aβ clearance, human recombinant (hr)AnxA1 exhibits protective effects on cerebrovascular integrity and cognitive function. In young 5xFAD mice, hrAnxA1 restored blood-brain barrier function, reduced vascular deficits, and modulated inflammation by increasing interleukin-10 and decreasing tumor necrosis factor-α levels (Ries et al., 2021). Additionally, hrAnxA1 treatment mitigated memory deficits and enhanced synaptic density in these mice, suggesting cognitive benefits. In Tau-P301L mice, hrAnxA1 stabilized vascular architecture, influenced tight junction distribution, and reduced Tau phosphorylation, likely through the RhoA-ROCK signaling pathway (Ries et al., 2021). Collectively, these findings position AnxA1 as a promising candidate for AD treatment, addressing cerebrovascular damage, inflammation, and cognitive decline.

Evidence suggests that the therapeutic potential of AnxA1 in AD extends beyond its roles in Aβ clearance and neuroprotection through FPR2 signaling, highlighting an intracellular function as a modulator of the NLR family pyrin domain containing 3 (NLRP3) inflammasome — an essential driver of neuroinflammation. In AD, the accumulation of Aβ and Tau triggers NLRP3 activation in microglia and astrocytes, leading to the production of active interleukin-1β and ASC specks, which perpetuates a self-feeding inflammatory cycle (Van Zeller et al., 2021). This cycle amplifies neuroinflammation, accelerates Aβ aggregation, and exacerbates neuronal damage, further impairing microglial phagocytic function (Koenigsknecht-Talboo and Landreth, 2005).

Therefore, AnxA1 modulation has emerged as a promising strategy to counteract NLRP3-driven inflammation across different pathological contexts. Although most AnxA1 actions are mediated via FPR2, evidence indicates that it can also regulate NLRP3 independently of this receptor (Galvão et al., 2020), adding complexity to its mechanisms of action. In the central nervous system, AnxA1-derived tripeptide effectively inhibited NLRP3 activation in the hippocampus, reducing microglial activation and preventing cognitive impairments following surgery, suggesting potential therapeutic applications in perioperative neurocognitive disorders and AD (Zhang et al., 2022). Furthermore, studies indicated that the absence of endogenous AnxA1 has been linked to excessive NLRP3 activation, intensifying inflammation (Sanches et al., 2020). Conversely, AnxA1 overexpression mitigates NLRP3 activation in arsenic-induced hepatic insulin resistance, highlighting its function as a metabolic regulator (Wu et al., 2022).

The ability of AnxA1 to modulate NLRP3 offers promising avenues for the development of targeted anti-inflammatory therapies, particularly for neurodegenerative diseases such as AD, where neuroinflammation plays a key role. The absence of endogenous AnxA1 has been associated with exacerbated neuroinflammation, suggesting that AnxA1 is a crucial endogenous regulator in mitigating inflammatory processes in AD. The impact of AnxA1 on dysfunctional synaptic circuits and the impaired phagocytic activity of microglia, a key factor in AD progression, should be examined. To further understand AnxA1 neuroprotective mechanisms, future studies should also focus on elucidating the AnxA1-mediated modulation of NLRP3 in a FPR2-independent manner. Furthermore, research should aim to identify the optimal duration and dosing strategies for AnxA1 therapy, as these parameters may be crucial for maximizing its clinical efficacy. Although recent findings have shown limited therapeutic benefits of hrAnxA1 at later stages of the disease (Ries et al., 2021), additional studies using alternative delivery approaches or combination therapies may help overcome this limitation. Long-term behavioral and mechanistic studies remain essential to clarify whether AnxA1-based interventions can offer sustained neuroprotection in chronic AD models. Addressing these gaps will be fundamental in advancing AnxA1-based therapies as a viable strategy for mitigating neuroinflammation and promoting neuroprotection in AD.

*The work in our laboratory was funded by the São Paulo Research Foundation (FAPESP; grant No. 2022/02327-6). LPSF is supported by the FAPESP scholarships (2021/00270-4 and 2024/05491-7). CDG is a research fellow of the Brazilian National Council for Scientific and Technological Development (CNPq).*

**Additional file:** *Open peer review report 1.*

OPEN PEER REVIEW REPORT 1
