# Supplementary material for: Annexin A1 in Alzheimer’s disease: A new therapeutic strategy focusing on neuroinflammation
Source: Neural Regen Res. 2025 Sep 3;21(6):2363–4. doi: 10.4103/NRR.NRR-D-25-00505 (PMC13211795; doi:10.4103/NRR.NRR-D-25-00505)
Supplement: Supplementary file 1 [file NRR-21-2363_Suppl1.pdf]

OPEN PEER REVIEW REPORT 1

Name of journal: Neural Regeneration Research
Manuscript NO: NRR-D-25-00505
Title: Annexin A1 in Alzheimer's disease: A new therapeutic strategy focusing on neuroinflammation
Reviewer's Name: Magdalena Sastre
Reviewer's country: UK

COMMENTS TO AUTHORS

The current perspective reviews the therapeutic potential of ANXA1 in Alzheimer's disease (AD). This is a topic that has raised interest in the last decade. Although in general the author/s have included the main findings so far, there are inaccuracies in the references and in some concepts:

1. The authors should expand the concept of the levels and expression of ANXA1 in AD. For instance, the paper from Park et al (2017), shows that ANXA1 is reduced in serum from AD patients. There are also previous publications (before Chua et al, 2022), describing the expression of ANXA1 in AD. For instance, it was published by Ries et al. (2016) that ANXA1 is increased in both AD brains and in animal models of AD. In addition, ANXA1 is upregulated in human microglia surrounding Aβ plaques (McArthur et al., Immunol. 2010;185:6317-28).
2. ANXA1 has also been involved in regulation of blood-brain barrier integrity in brain endothelial cells and can restore functionality in ANXA1 knockout mice. The effect of ANXA1 on the blood-brain barrier appears to be exerted via activation of formyl peptide receptor-2 (FPR2), which is expressed on brain microvascular endothelial cells. Particularly, ANXA1 binding to FPR2 inhibits RhoA signalling, leading to cytoskeletal stabilization, ultimately decreasing paracellular permeability (Cristante et al., Proc Natl Acad Sci USA. 2013;110:832-841).
3. The correct reference for the treatment of TauP301L mice with ANXA1 in the first paragraph of page 2 is Ries et al, 2021 and not Park et al., 2017.
4. Some of the references regarding the role of ANXA1 regulating the inflammasome component NLRP3 have little to do with Alzheimer's disease and perhaps should be removed.
5. Some of the future perspectives have already been explored, such as the role of ANXA1 at chronic stages of AD. It was already shown by Ries et al., 2021 that in older AD mice there is no benefit of the treatment.
6. Some of the schematics in figure 1 are not correct. The classification of microglia phenotypic states M1 vs M2 is not accepted any more (Paolicelli et al, Neuron. 2022 Nov 2;110(21):3458-3483). The effect of ANXA1 on BBB is not mediated by astrocytes, as reported by Cristante et al., 2013.
